# Investigation of physical and functional impairments experienced by people with active tuberculosis infection: A feasibility pilot study

**DOI:** 10.4102/ajod.v8i0.515

**Published:** 2019-08-13

**Authors:** Heleen van Aswegen, Ronel Roos, Melanie McCree, Samantha Quinn, Mervyn Mer

**Affiliations:** 1 Department of Physiotherapy, Faculty of Health Sciences, University of the Witwatersrand, Johannesburg, South Africa; 2 Wits-University of Queensland Critical Care Infection Collaboration Group, Faculty of Health Sciences, University of the Witwatersrand, Johannesburg, South Africa; 3 Department of Medicine, Divisions of Critical Care and Pulmonology, Charlotte Maxeke Johannesburg Academic Hospital, Faculty of Health Sciences, University of the Witwatersrand, Johannesburg, South Africa

**Keywords:** tuberculosis, physical function, muscle cross-sectional area, HIV, muscle mass

## Abstract

**Background:**

Tuberculosis (TB) remains a significant healthcare problem. Understanding physical and functional impairments that patients with active TB present with at the time of diagnosis and how these impairments change over time while they receive anti-TB therapy is important in developing appropriate rehabilitation programmes to optimise patients’ recovery.

**Objectives:**

The aim of this study was to assess the acceptability, implementation and practicality of conducting a prospective, observational and longitudinal trial to describe physical and functional impairments of patients with active TB.

**Method:**

A feasibility pilot study was performed. Patients with acute pulmonary TB admitted to an urban quaternary-level hospital were recruited. Physical (muscle architecture, mass and power, balance, and breathlessness) and functional (exercise capacity) outcomes were assessed in hospital, and at 6 weeks and 6 months post-discharge. Descriptive statistics were used to analyse the data.

**Results:**

High dropout (*n* = 5; 41.7%) and mortality (*n* = 4; 33.3%) rates were observed. Limitations identified regarding study feasibility included participant recruitment rate, equipment availability and suitability of outcome measures. Participants’ mean age was 31.5 (9.1) years and the majority were human immunodeficiency virus (HIV) positive (*n* = 9; 75%). Non-significant changes in muscle architecture and power were observed over 6 months. Balance impairment was highlighted when vision was removed during testing. Some improvements in 6-minute walk test distance were observed between hospitalisation and 6 months.

**Conclusion:**

Success of a longitudinal observational trial is dependent on securing adequate funding to address limitations observed related to equipment availability, staffing levels, participant recruitment from additional study sites and participant follow-up at community level. Participants’ physical and functional recovery during anti-TB therapy seems to be limited by neuromusculoskeletal factors.

## Introduction

Tuberculosis (TB) remains a very significant and pertinent healthcare problem. It is currently the leading cause of mortality associated with a single infectious pathogen globally, and one of the top 10 causes of death worldwide (WHO 2018). In 2016, close to 10.5 million people fell ill with TB and 1.7 million people succumbed to the disease. Patients with human immunodeficiency virus (HIV) disease are at substantially increased risk of acquiring TB, with 40% of all HIV deaths related to TB (WHO 2018). In South Africa, TB is the overall number one cause of mortality (Statistics South Africa 2015), and scores of patients with TB are admitted to hospitals and seen in clinics on a daily basis. Many of these patients are, however, successfully treated for TB and survive to hospital or clinic discharge.

Over the past several years, increasing attention has been focused on the *sequelae* [a condition which is the consequence of a previous disease or injury] that survivors of acute illness may experience, such as impairments in functional status and quality of life (QOL) (Azoulay et al. 2017; Biehl et al. 2015; Connolly et al. 2015; Elliott et al. 2011; Fan 2012). In addition, there may be serious social and economic consequences and ramifications (Griffiths et al. 2013). These elements are frequently overlooked (Gaudry et al. 2017). Patients with active TB infection report deficits in their physical and mental well-being, and it is suggested that symptoms such as fatigue, loss of weight, loss of appetite, fever and bodily pain may impact their ability to function in society (Atif et al. 2014). Patients who receive anti-TB therapy undergo prolonged treatment (up to 9 months) and are reported to experience anxiety and fear of social exclusion as they are viewed as being an infection source in the community that they live in (Guo et al. 2009; Kibrisli et al. 2015). The impact of pulmonary TB on patients’ QOL has been widely reported. Patients with TB experience low levels of physical and mental health-related aspects of QOL at the time of disease diagnosis (Atif et al. 2014; Babikako et al. 2010; Guo et al. 2009; Kastien-Hilka et al. 2017; Louw et al. 2012). Anti-TB therapy significantly improves all aspects of QOL (Babikako et al. 2010; Kastien-Hilka et al. 2017); however, after successful completion of anti-TB therapy, these patients’ health-related QOL remains significantly lower than that of the general population (Atif et al. 2014; Guo et al. 2009). Other factors such as level of dyspnoea, low body mass index and unemployment have been related to decreased levels of physical and mental health-related QOL in patients with pulmonary TB (Atif et al. 2014; Kastien-Hilka et al. 2017; Masumoto et al. 2014). Furthermore, functional impairments, such as balance and gait abnormalities, have been linked with HIV seropositivity (Bauer et al. 2005; Fama et al. 2007; Trenkwalder et al. 1992).

### Problem statement

The high incidence and prevalence of TB and HIV in South Africa necessitate research into the effect of these diseases on patients’ physical and functional status. Research shows that people with TB suffer from long-term limitations in physical and mental health-related aspects of QOL as well as limitations in their exercise capacity, often because of compromised lung function (Cole et al. 2016; Guessogo et al. 2016; Guo et al. 2009; Kastien-Hilka et al. 2017; Sivaranjini, Vanamail & Eason 2010). The impact of the symptoms of TB on patients’ muscle function in relation to muscle architecture, mass and power at the time of disease diagnosis and changes in these parameters during anti-TB therapy has not been quantified to date. In addition, the impact that these changes in muscle architecture, mass and power may have on patients’ ability to balance and their exercise endurance has not been reported previously.

### Rationale of the study

The evidence that is currently available on limitations in exercise capacity and pulmonary function of South African patients with pulmonary TB reports on patients who have been on anti-TB therapy for several months prior to testing (Cole et al 2016; De Grass, Manie & Amosun 2014). Understanding and quantifying the level of physical (level of breathlessness, muscle architecture and mass, muscle power, balance) and functional (exercise capacity) impairments that patients with active TB infection present with at the time of disease diagnosis may be useful to physiotherapists in developing appropriate rehabilitation programmes for these patients during their hospital stay and after discharge to optimise their recovery to a comparable level with that of a healthy general population at the time of completion of anti-TB therapy. Clinical trials, using a longitudinal design, are often limited by high patient dropout rates after hospital discharge (Abshire et al. 2017; Roos, Myezwa & Van Aswegen 2013; Van Aswegen et al. 2010). This feasibility study was, therefore, conducted to assess the acceptability, implementation and practicality (Bowen et al. 2009) of conducting a prospective, observational and longitudinal trial in an urban setting of a developing country to describe physical and functional impairments of patients at the time of active TB diagnosis and to evaluate changes in these outcomes over the 6-month period of anti-TB therapy.

## Research method

### Design

A feasibility pilot study was conducted to test the study objectives.

### Subjects and setting

Patients admitted to the infectious diseases ward or the general intensive care unit (ICU) at Charlotte Maxeke Johannesburg Academic Hospital (CMJAH) with active TB infection were considered for participation in this project. Charlotte Maxeke Johannesburg Academic Hospital is an accredited central university-affiliated quaternary-level government hospital in Johannesburg that offers a range of specialist inpatient and outpatient services to residents across Gauteng and neighbouring provinces. Diagnosis of TB was made through patient clinical presentation, radiological investigations and on-site access to GeneXpert for confirmation of pulmonary TB. A sputum sample was sent for GeneXpert for all patients with suspected TB and results were obtained on the day of testing. Urine lipoarabinomannan (LAM) testing and tissue biopsy were performed for select patients when deemed appropriate to assist with diagnosis. Physiotherapy service provision at CMJAH involved inpatient management only and no post-discharge physiotherapy services were offered to patients with TB without neurological symptoms. Post-discharge follow-up of participants was done at the Physiotherapy Department Movement Laboratory on the University of the Witwatersrand Education Campus in Parktown, which is situated opposite CMJAH.

A consecutive sampling method was used to recruit participants for this feasibility pilot study. Both male and female patients 18 years or older, with or without HIV seropositivity, who were diagnosed with active TB infection were approached regarding possible participation. Subjects were not considered for participation if they had TB meningitis, were unable to stand and walk without assistance, had recent neurosurgery or head injury with loss of consciousness for more than 10 min, recent orthopaedic injury to the lower limbs, amputation, diagnosed major comorbidity (rheumatic disease, malignancy, psychiatric disease, cirrhosis or epilepsy, chronic obstructive pulmonary disease), ocular disease or vestibular disorders.

The study was conducted over a 15-month period and all eligible patients who provided consent were included. Participant recruitment for baseline hospital assessment was performed over a 12-month period (May 2016–May 2017). Six-month follow-up assessments for all participants were completed by August 2017.

### Instrumentation

Two of the researchers (H.v.A. and S.Q.) performed testing on all participants. Prior to the pilot study these researchers underwent training in the use of all of the instrumentation listed below.

#### Balance

The Berg balance scale was used to assess participants’ balance. It is a 14-item objective measure that assesses static and dynamic balance and risk for falls in adults. Item-level scores range from 0 to 4, and scores for each item are added together to determine each participant’s score achieved out of a maximum score of 56. It is a reliable and valid tool for the assessment of balance in a variety of populations, including those with respiratory diseases (Oliveira et al. 2013).

Two additional balance tests with the addition or removal of vision as a balance strategy were included. These tests were standing on one leg with the hip held at 90° (marching position) for 10 s, and single-leg squat. These tests were added as it was anticipated that there might be a ceiling effect of the Berg balance scale scores in this patient population.

The timed up-and-go (TUG) test was used to assess functional balance. It assesses a participant’s ability to stand up from a chair, walk 3 m, turn around, walk back to the chair and sit down. It also assesses the time in which these activities are completed. Duration of greater than 13.5 s predicts a high risk of falls. The TUG test is a widely used reliable and valid tool for the assessment of functional balance (Steffen, Hacker & Mollinger 2002).

#### Exercise capacity

The 6-minute walk test (6MWT) was used to determine participants’ exercise capacity. It is a functional walk test that assesses the distance a person can walk on a flat surface in 6 min using a 30-m track. Instructions on performance of the 6MWT, as outlined by the American Thoracic Society guidelines (2002), were implemented. The 6MWT is a well-known reliable and valid tool for the assessment of exercise capacity in a wide range of patient populations, including those who suffer from respiratory diseases (Sivaranjini et al. 2010). The distance a person achieves on the 6MWT should be interpreted against their predicted distance achieved with regard to age and gender, using reference equations for a healthy population. There are currently no reference equations for healthy South Africans. Therefore, for this study, the reference equation published by Gibbons et al. (2001) for healthy adults aged 22–68 years was used to calculate predicted distance achieved on the 6MWT.

#### Level of dyspnoea

Level of dyspnoea was assessed using the modified Borg (0–10) scale. The higher the score, the more limited a participant’s function would be as a result of dyspnoea. It is a reliable and valid tool for the measurement of level of dyspnoea in patients with respiratory diseases (Kendrick, Baxi & Smith 2000).

#### Muscle architecture and mass

**FIGURE 1:**
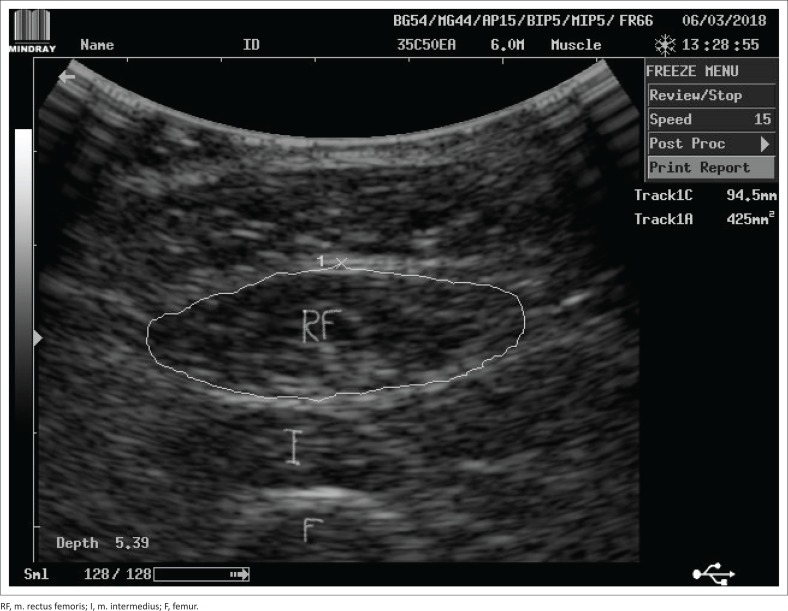
Example of measurement of cross-sectional area of the rectus femoris muscle of a healthy individual using on-screen callipers of the ultrasound diagnostic imaging system.

**FIGURE 2:**
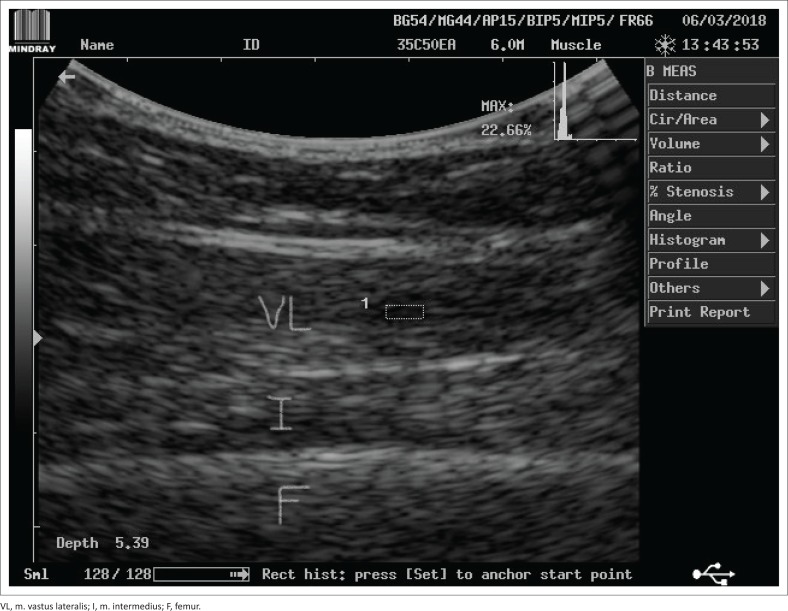
Example of measurement of echo intensity of the vastus lateralis muscle of a healthy individual using on-screen callipers of the ultrasound diagnostic imaging system.

Ultrasound imaging was used to assess muscle cross-sectional area (CSA), thickness (mass) and echo intensity. A DP-6600 model digital ultrasonic diagnostic imaging system (Shenzhen Mindray Bio-Medical Electronics Co., Ltd.) with a 5-MHz curvilinear transducer and a large footprint (≥60 mm) was used. B-mode was selected to image each participant’s rectus femoris and vastus lateralis muscles of their dominant leg. These muscles form part of the quadriceps muscle group and play an important role in facilitating hip flexion and knee extension actions, while a person performs physical activities such as walking, running and jumping. Measurements were done with on-screen callipers. Muscle CSA was measured (mm^2^) in the transverse image plane using the on-screen calliper to manually outline the inner echogenic line that represented the fascia around the rectus femoris muscle (Figure 1). Muscle thickness was measured (mm) as the distance between the superficial and the deep fascia at the widest distance of the rectus femoris muscle. Echo intensity (measured as %) was obtained in the longitudinal ultrasound image plane, where a region of interest was selected in the vastus lateralis muscle without any bone or surrounding fascia (Figure 2). The validity of ultrasound imaging as an effective technique to detect musculoskeletal disorders and muscle tissue trauma was described previously by Pillen (2010) and Strasser et al. (2013).

#### Muscle power

The Medical Research Council sum score (MRC-SS) was used to assess muscle power. The power of three predetermined upper extremity and three predetermined lower extremity muscle groups is assessed bilaterally on a scale of 0–5, where 0 represents no visible muscle contraction and 5 represents normal muscle power (against gravity and resistance). The maximum score that can be achieved is 60 with normal muscle power reflected by a score closer to 60/60. The MRC-SS is a widely used reliable and valid tool to assess muscle power in various patient populations, including patients with acute illness (Connolly et al. 2013; Vanpee et al. 2014).

### Procedure

Patient admissions into the infectious diseases ward and the general ICU were monitored by one of the researchers (M.M.C.). Potential participants were identified by the nurse in charge of the unit. The researcher (M.M.C.), in the presence of the charge nurse, approached these potential participants to screen them for eligibility against the inclusion and exclusion criteria as soon as their condition had stabilised. Detailed explanation of all tests and procedures involved were provided to participants. They were given 24 h to consider participation before written consent was obtained (by M.M.C.). Demographic information (age, gender, date of admission, date of commencement of anti-TB medication, etc.) was obtained from the medical file of each consenting participant. A short interview was held (by M.M.C.) with each participant, while they were seated comfortably in bed or in a chair, to obtain additional information (e.g. diagnosed comorbidities, living conditions, employment status, level of physical activity, smoking history and alcohol use prior to admission). The information collected from each participant was used as a baseline assessment.

At the start of the assessment, each participants’ level of dyspnoea was assessed with the modified Borg scale and recorded. For ultrasound imaging, participants were placed in a supine position on their hospital bed with both knees extended but relaxed and toes pointing to the ceiling (neutral rotation). The scanning site for the rectus femoris muscle was determined by identifying the mid-point on the line from the greater trochanter of the femur to the knee joint line. A generous amount of ultrasound contact gel was placed on the skin surface over the scanning site before the transducer was placed over this site applying minimal pressure. The transducer was placed perpendicular to the femur to visualise the rectus femoris muscle and obtain baseline measurements for muscle CSA and thickness. The transducer was placed parallel to the femur to visualise the vastus lateralis muscle to obtain baseline measurements for echo intensity. Two measurements for each outcome were taken and the average of each of these measurements was recorded (Pillen 2010; Strasser et al. 2013).

Muscle power was assessed with participants sitting upright over the edge of the hospital bed. The MRC-SS was used to assess power of shoulder abduction, elbow flexion and wrist extension movements of bilateral upper extremities. Hip flexion, knee extension and ankle dorsiflexion power were assessed for both the right and left lower extremities. Because the MRC-SS involves volitional muscle testing, strong verbal encouragement was given to each participant during the test (Connolly et al. 2013). Scores obtained for each muscle action were recorded.

Thereafter participants were given an opportunity to rest (approximately 3 min) before their balance was assessed with the Berg balance scale and the two additional balance tests. This involved participants performing various predetermined activities in sitting and standing by the bedside to assess static and dynamic balance and risk of falls. Next, functional balance was assessed with the TUG test. Participants were asked to sit in an upright chair by their bedside. A stopwatch was used to measure the total duration for participants to stand up from the chair, cover the 3-m distance, turn around, return to the chair and sit down. The time to completion of the test was recorded and a note was made of any assistive devices that the participant used during the test.

Lastly, the 6MWT was performed to obtain information on distance walked and percentage of predicted distance achieved. Prior to the test, participants were allowed to rest seated in a chair for 5 min. Their baseline heart rate, blood pressure and peripheral oxygen saturation values were measured (SSEM Mthembu Medical noninvasive blood pressure patient monitoring device with SpO_2_) and recorded. Thereafter the test was commenced. Participants were instructed to walk at a comfortable pace of moderate intensity to ensure as much distance as possible is covered and that an adequate exercise response was obtained during the test. Encouragement to participants during the walk test was given strictly according to the prescribed guidelines (American Thoracic Society 2002). Walk distance achieved, changes in heart rate, blood pressure and peripheral oxygen saturation together with post-exercise level of dyspnoea were recorded immediately after the test and again after 5 min of rest.

At the end of the session, the researcher gave each participant an appointment date and time for the next assessment at 6 weeks post-discharge, together with her contact details and a map to the University of the Witwatersrand Education Campus Physiotherapy Department to make the location of the test venue clear. The researcher confirmed each participant’s contact details with them as well as contact details of their relatives or neighbours to allow for post-discharge reminders to be sent to them regarding their next appointment dates and times. Two weeks prior to their next appointment they were telephoned by the same researcher to remind them of the appointment and 2 days before the appointment they received a text message reminder in an attempt to improve compliance.

At the 6 weeks and 6 months follow-up appointments, one of the researchers (H.v.A. or S.Q.) verified each participant’s details and demographic information with them and changes in details were recorded. All outcome measures assessed during hospitalisation were repeated at both of these assessment points.

### Data analysis

The data obtained were nominal, ordinal and ratio in nature. Data analysis was performed using IBM® SPSS version 24 for Windows software and intention-to-treat analysis was done. Missing data for those who were still alive but dropped out of the trial were managed through last observation carried forward. Descriptive statistics were used to present the data and normality of data was tested using the Shapiro–Wilk test. Categorical data were summarised as frequencies and percentages. Continuous data were presented as mean, and standard deviations (SD) for normally distributed data and median and interquartile range (IQR) for non-normally distributed data. To determine changes in outcomes over time, ANOVA with repeated measures with a Greenhouse–Geisser correction was performed using Bonferroni confidence interval adjustment. The level of significance was set at ≤0.05 with a 95% confidence interval (CI). No regression analysis was performed because of the small sample size.

### Ethical considerations

Permission to conduct the study was obtained from the University of the Witwatersrand Human Research Ethics (Medical) committee (certificate number: M150857) and the hospital review board. The study was registered with the South African National Clinical Trials Registry (trial number: DOH-27-1215-5241). The principles for conducting research on human subjects, as outlined in the Declaration of Helsinki, were adhered to throughout the duration of the trial.

## Results

**FIGURE 3:**
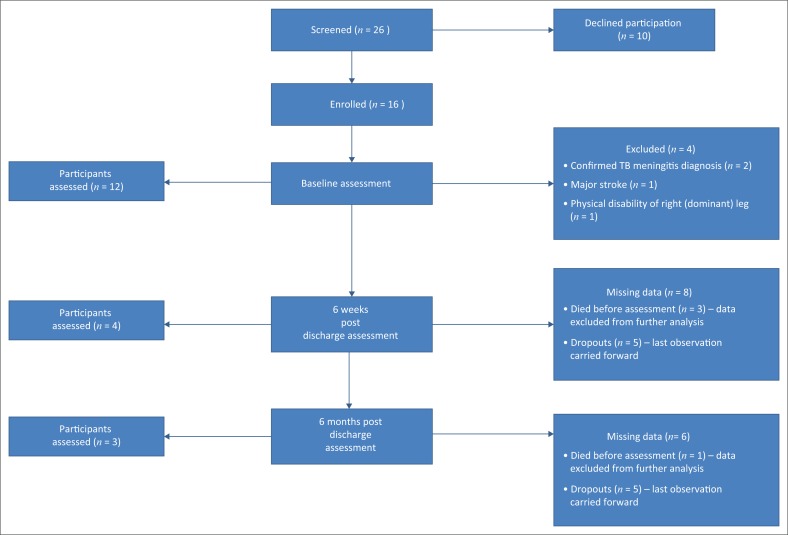
Participants recruitment and flow throughout the study period.

Sixteen participants were enrolled into the study during the 12 months of the recruitment period. Figure 3 summarises participant recruitment and flow through the study. Some participants were excluded at baseline assessment because of confirmed diagnosis of TB meningitis, stroke or physical disability. Four of the 12 participants died after hospital discharge because of causes unrelated to the study procedures, and this resulted in an overall mortality rate of 33.3%. A high dropout rate of 41.7% (*n* = 5) was observed over the 6 months of the observation period.

The mean age of participants (*n* = 12) was 31.5 (9.1) years. Reported symptoms experienced before admission varied widely and included shortness of breath, vomiting, headaches, feeling disoriented, tiredness and night sweats. Only two participants were admitted to ICU and had a mean duration of stay of 1.1 (2.3) days. One was lost to follow-up after hospital discharge and the other died before the 6 weeks follow-up assessment. Participants were commenced on anti-TB therapy on average within 2 days from admission. Most were managed with first-line anti-TB therapy (Rifafour and Ethambutol), but two presented with drug-resistant TB and were managed with Ethionamide, Isoniazid and Pyrazinamide. The median duration of hospitalisation for the whole group (*n* = 12) was 6.5 (IQR 4.8–16.5) days. Table 1 summarises the demographic information obtained for the study. The majority of participants were female (*n* = 10, 83.3%) and most participants (*n* = 9, 75%) presented with pulmonary TB and were HIV seropositive (*n* = 9, 75%). The majority of participants (*n* = 8, 66.7%) did not partake in exercise prior to admission and none of the participants reported a history of smoking. Few participants had returned to employment at 6 months.

**TABLE 1:** Demographic information of the study cohort (*n* = 12).

Demographics	Frequency	Per cent
**Gender**		
Female	10	83.3
Male	2	16.7
**Comorbidities**		
Asthma	1	8.3
Congestive heart failure	2	16.7
Diabetes mellitus	1	8.3
Hearing impairment	1	8.3
HIV	9	75.0
None	3	25.0
**Marital status**		
Single	11	91.7
Live-in partner	1	8.3
**Employment status on admission**		
Yes	8	66.7
No	4	33.3
**Employment status at 6 weeks after discharge (*n* = 9)**		
Yes	4	44.4
No	5	55.6
**Employment status at 6 months after discharge (*n* = 8)**		
Yes	3	37.5
No	5	62.5
**Type of TB**		
Pulmonary	9	75.0
Disseminated	2	16.7
Multidrug resistant	1	8.3
**Exercise performed prior to admission**		
Yes	4	33.3
No	8	66.7
**Exercise performed at 6 weeks after discharge (*n* = 9)**		
Yes	4	44.4
No	5	55.6
**Exercise performed at 6 months after discharge (*n* = 8)**		
Yes	3	37.5
No	5	62.5
**Smoking status**		
Yes	0	0.0
No	12	100.0
**Alcohol use status on admission**		
Yes	2	16.7
No	10	83.3
**Alcohol use at 6 weeks after discharge (*n* = 9)**		
Yes	2	22.2
No	7	77.8
**Alcohol use at 6 months after discharge (*n* = 8)**		
Yes	2	25.0
No	6	75.0

HIV, human immunodeficiency virus; TB, tuberculosis.

### Muscle architecture, mass and power

Findings for muscle CSA, thickness and echo intensity are summarised in Table 2. Results are presented for nine participants only during hospitalisation as the ultrasound machine was unavailable for three participants’ assessment because of another study for which it was used at the time. The greatest change in mean muscle CSA (110.3 mm^2^) occurred between the 6 weeks and 6 months of assessments, whereas the greatest changes in mean muscle thickness (2.3 mm) and mean echo intensity (5.2%) occurred between the hospital assessment and 6 weeks follow-up. Changes observed for these parameters over the 6-month period were, however, not significant (CSA: *p* = 0.2; thickness: *p* = 0.19; echo intensity: *p* = 0.18).

**TABLE 2:** Results for rectus femoris and vastus lateralis muscles with respect to muscle cross-sectional area, thickness and echo intensity using diagnostic ultrasound imaging.

Variable	Mean	SD	95% confidence interval	Range
**Cross-sectional area (mm^2^)**				
Hospitalisation (*n* = 9)	195.9	137.7	−23.2–415.1	109.9–400.0
6 weeks (*n* = 7)	282.5	149.8	44.2–520.9	109.9–415.5
6 months (*n* = 6)	392.8	200.5	73.8–711.9	109.9–565.5
**Thickness (mm)**				
Hospitalisation (*n* = 9)	6.2	3.0	2.4–9.9	4.0–11.3
6 weeks (*n* = 7)	8.5	4.0	3.5–13.4	4.2–13.4
6 months (*n* = 6)	9.8	5.0	3.6–16.1	4.2–14.6
**Echo intensity (%)**				
Hospitalisation (*n* = 9)	23.3	5.4	16.6–30.0	19.3–31.8
6 weeks (*n* = 7)	18.1	2.6	14.9–21.3	14.0–20.6
6 months (*n* = 6)	18.9	1.6	16.9–20.9	16.2–20.6

SD, standard deviation.

The mean MRC-SS during hospitalisation for this cohort (*n* = 10) was 51.2 (1.7; 95% CI, 49.4–53; range: 49–53), which changed to 52 (3.2; 95% CI, 48.7–55.3; range: 49–56) at 6 weeks (*n* = 7), with a mean MRC-SS of 52.8 (4.1; 95% CI, 48.5–57.2; range: 49–58) at 6 months post-discharge (*n* = 6). A mean change in muscle power of 0.8 was observed between hospital assessment and 6 weeks follow-up and between 6 weeks and 6 months post-discharge, respectively. The changes observed in MRC-SS over the 6-month period were not significant (*p* = 0.3).

### Balance assessment

Balance was assessed with the Berg balance scale and the TUG test. The mean Berg balance scores were 53.7 (2.1; 95% CI, 51.5–55.8; range: 51–56; *n* = 10), 54.5 (1.9; 95% CI, 52.5–56.5; range: 51–56; *n* = 7) and 54.7 (2; 95% CI, 52.6–56.7; range: 51–56; *n* = 6), respectively, at the three assessment points. Four of the 10 participants (40%) managed to achieve the highest score (56/56) at hospital assessment. The greatest change in mean scores (0.8) was observed between the hospital assessment and 6 weeks follow-up. The changes observed in mean Berg scores over the 6-month period were not significant (*p* = 0.18).

During hospitalisation, participants completed the TUG test in a mean duration of 10.2 (1.8; 95% CI, 8.3–12.1; range: 7.4–11.9; *n* = 9) s. At 6 weeks they completed the test in a mean duration of 9.5 (2; 95% CI, 7.4–11.7; range: 7.2–11.9; *n* = 7) s and at 6 months in 9.8 (2.4; 95% CI, 7.3–12.4; range: 5.3–11.9; *n* = 6) s. None of the participants used an assistive device during the TUG test assessment at any of the three assessment points. A high risk of falls was identified in only one participant during hospitalisation and in no participants at 6 weeks or 6 months after discharge. The greatest change in mean TUG test duration (0.7 s) was observed between hospital assessment and 6 weeks. The changes observed in TUG test duration over the 6-month period were not significant (*p* = 0.36).

Results for the additional balance tests performed with eyes open and eyes closed are presented in Table 3. It was noted that participants’ ability to balance was drastically decreased when they were asked to perform each of the two tests with their eyes closed. This was observed at all three assessment points.

**TABLE 3:** Additional balance test results with eyes open and eyes closed.

Tests performed on dominant leg	Hospital (*n* = 8)	Per cent	Six weeks (*n* = 6)	Per cent	Six months (*n* = 5)	Per cent
**Stand on one leg with hip at 90° (march position) for 10 s, eyes open**						
Unable to perform	2	25	0	0	0	0
Able to perform	6	75	6	100	5	100
**Stand on one leg with hip at 90° (march position) for 10 s, eyes closed**						
Unable to perform	7	86	4	66.7	2	40
Able to perform	1	14	2	33.3	3	60
**Single-leg squat, eyes open**						
Unable to perform	5	63	2	33.3	2	40
Able to perform with poor form	3	37	2	33.3	0	0
Able to perform with good form	0	0	2	33.4	3	60
**Single-leg squat, eyes closed**						
Unable to perform	7	86	3	50	3	60
Able to perform with poor form	1	14	1	16.7	1	20
Able to perform with good form	0	0	2	33.3	1	20

### Level of dyspnoea

The mean Borg score during hospitalisation (*n* = 10) was 1.5 (1.4; 95% CI, 0.1–2.3; range: 0–3), which represented ‘very slight’ and ‘slight’ breathlessness. The mean score changed to 1 (0.9; 95% CI, 0.1–1.9; range: 0–2) at 6 weeks (*n* = 7), and 1.1 (1.2; 95% CI, −0.2 to 2.3; range: 0–3) at 6 months (*n* = 6) post-discharge, which represents ‘very slight’ breathlessness. The greatest change in mean Borg scores (0.5) was between hospitalisation and the 6 weeks follow-up assessment. The changes observed in modified Borg scores over the 6-month period were not significant (*p* = 0.31).

### Exercise capacity

The 6MWT was performed over a 10-m distance on the hospital ward because of lack of open space available. At 6 weeks and 6 months, a 30-m track was used in the Physiotherapy Department as recommended by the American Thoracic Society (2002) guidelines. Participants’ results are summarised in Table 4. The greatest change in mean distance walked (103.7 m) was observed between the hospital assessment and the 6 weeks follow-up assessment. There was a reduction of 62.1 m in mean distance walked between the 6 weeks and 6 months of assessments. The greatest improvement in percentage of predicted distance achieved (14.2%) was observed between hospitalisation and the 6 weeks follow-up assessment. Between the 6 weeks and 6 months of assessments, participants achieved less of the percentage of predicted distance walked (8.4%) on the 6MWT. The changes observed in distance walked and in percentage of predicted distance achieved over the 6-month period were not significant (*p* = 0.13 and *p* = 0.14, respectively). There was reduction in mean peripheral oxygen saturation levels between rest and immediately after completion of the 6MWT of −0.14% at hospital assessment. This changed to −3.1% at 6 weeks and −2% at 6 months. After 5-min rest, there was an improvement in mean peripheral oxygen saturation levels of 4.3%, 3.7% and 3% at the three assessment points, respectively.

**TABLE 4:** Results for exercise endurance as measured with the six-minute walk test.

Variable	Mean	Standard deviation	95% confidence interval	Range
**Distance walked (m)**				
Hospitalisation (*n* = 7)	315.8	157.8	119.8–511.8	60–495.0
6 weeks (*n* = 6)	419.5	235.6	127.0–712	60–645.0
6 months (*n* = 5)	357.4	180.3	133.5–581.3	60–536.1
**Percentage of predicted distance achieved**				
Hospitalisation (*n* = 7)	43.2	20.6	17.7–68.8	8.6–63.8
6 weeks (*n* = 6)	57.4	31.3	18.6–96.3	8.6–85.8
6 months (*n* = 5)	49.0	23.8	19.5–78.6	8.6–69.1

## Discussion

This study was conducted to assess the acceptability, implementation and practicality of conducting a prospective, observational and longitudinal trial in an urban quaternary-level hospital setting to evaluate physical and functional impairments of patients diagnosed with active TB infection. In addition, preliminary findings on changes in muscle architecture, mass and power, level of dyspnoea, balance and exercise capacity over the study period are presented.

Acceptability in feasibility studies refers to how participants in a trial react to an intervention (Bowen et al. 2009). Participants in this study were keen to perform the tests used to assess their levels of physical and functional impairments while in hospital. No adverse events occurred as a result of any of the test procedures. A high dropout rate (*n* = 5, 41.7%) was, however, observed over the 6 months follow-up period. Attempts made by the researchers to prevent loss to follow-up such as written information given to participants regarding appointment dates and times and venue prior to discharge, and reminder telephone calls and text messaging seemed to be unsuccessful in some cases. Some participants provided contact details during the hospital assessment session that proved to be incorrect after they were discharged as their mobile phone numbers were not in service. In some cases the calls made went unanswered. During telephonic conversation, some participants who dropped out of the study after hospital discharge commented that they did not have the financial means to return to the test venue for follow-up assessments. This was stated despite the fact that funding was in place to reimburse participants for their travel costs from home to the Wits University Education Campus for follow-up assessment. This information was verbalised to them prior to hospital discharge. Others stated that they felt too weak to endure the travel journey from their homes to the test venue.

High loss to follow-up experienced in rehabilitation-based trials in Gauteng Province of South Africa is not unusual (Roos et al. 2013; Van Aartsen & Van Aswegen 2018; Van Aswegen et al. 2010). Roos et al. (2013), when screening physical activity levels and physical activity preferences of relatively healthy HIV individuals attending an HIV roll-out centre in Johannesburg, observed a dropout rate of 27.3%. In contrast, Van Aswegen et al. (2010), when evaluating the effect of penetrating trunk trauma and mechanical ventilation on the recovery of adult survivors after hospital discharge, noted a dropout rate of 46% in their study participants who required less than 5 days of mechanical ventilation during hospital stay. Van Aartsen and Van Aswegen (2018) reported a 54.2% dropout rate over a 6-month period while investigating changes in biopsychosocial outcomes in a mixed cohort of ICU survivors in South Africa. The reasons for participant dropout listed by the abovementioned authors are similar to those experienced in this feasibility study. The strategies employed during this study to retain study participants after hospital discharge are supported in the literature (Abshire et al. 2017). Those participants who completed the feasibility study verbalised a positive attitude towards their participation as they valued seeing small improvements in their own physical and functional abilities over the 6-month period. Follow-up of participants at local community clinics or at their homes, instead of at a centralised urban venue, would reduce the distance travelled by participants and could present a possible solution to overcoming the financial constraints and levels of fatigue that some participants experienced.

Implementation in feasibility research investigates the extent, likelihood and manner in which an intervention can be fully implemented as planned (Bowen et al. 2009). A large portion of participants achieved the highest score on the Berg balance scale during initial hospital assessment. An instrument presents with a ceiling effect if more than 15% of participants achieve the highest possible score (Terwee et al. 2007). This suggests that the Berg balance scale might not be sensitive enough to accurately detect balance disturbances in this patient population; however, the sample size for this study was small and therefore findings should be interpreted with caution. Alternatively, the Y-balance test might be more appropriate for this patient population as it assesses dynamic balance. Its reliability and effectiveness has been demonstrated in middle-aged and older healthy females (Lee et al. 2015), but not in patients with TB.

The walking distance available in the ward setting of this quaternary hospital for the 6MWT was problematic. There was only a 10-m distance of relatively open free space available at the back of the ward over which the walk test could be performed. This might have led to sub-optimal participant performance because of the frequent turning enforced by the shorter track (Van Aartsen & Van Aswegen 2018). Consideration should, thus, be given to using an alternative test to assess participants’ exercise capacity in hospital such as the 3-min step test. This exercise test can be performed by a patient’s bedside and might give a more accurate assessment of exercise capacity than the 6MWT during hospitalisation. As few participants in this study presented with balance disturbances at their initial assessment in hospital, the 3-min step test may be a suitable alternative for the assessment of exercise capacity.

Practicality in feasibility research explores the extent to which an intervention can be delivered when resources, time and commitment may be constrained in some way (Bowen et al. 2009). During the 12 months of recruitment period, of the 26 patients screened, only 16 patients agreed to participate in the study. The rate of recruitment from this single study site was slower than initially anticipated. Consideration should, thus, be given to involving more hospital sites from which potential participants can be recruited for a longitudinal study. The relatively high post-discharge mortality rate observed during this feasibility study (33.3%) was concerning as it creates challenges for participant retention when using a longitudinal study design. This would be another motivating factor for involving more than one study site for longitudinal trials. Limited availability of the ultrasound imaging equipment was problematic and therefore purchase of one additional device is recommended.

The young cohort in this study presented with small non-significant improvements in muscle power over the 6-month observation period. Skeletal muscle power is dependent on skeletal muscle mass, composition and architecture (Strasser et al. 2013). Higher echo intensity reflects more intramuscular fat deposition and fibrous tissue formation in healthy older people (Strasser et al. 2013). The greatest increase in muscle thickness and power and decrease in muscle echo intensity for this cohort occurred between hospital assessment and assessment at 6 weeks. An increase in physical activity is associated with decreased muscle echo intensity and increased muscle thickness in patients recovering from critical illness (Parry et al. 2015). This is confirmed by the present study’s physical function capacity findings of an increase in distance walked on the 6MWT between hospital assessment and assessment at 6 weeks post-discharge. Most participants were employed prior to admission, but few had returned to employment following discharge. Few participants reported that they exercised regularly prior to admission and few had taken up exercise activity by 6 months after discharge. The changes in muscle architecture, mass and power observed over the 6-month period were not significant as participants seemed to lead a sedentary lifestyle; however, these findings can also be attributed to the small sample size.

There was a gradual increase in distance walked and percentage of predicted distance achieved on the 6MWT over the 6-month period compared with baseline findings. However, the mean distance achieved at 6 months was still significantly shorter than that reported for a healthy South African cohort (*n* = 40; 662 ± 78.3 m) (Van Aswegen et al. 2010). The reduction in peripheral oxygen saturation observed immediately after the 6MWT could imply that participants suffered a certain degree of exercise hypoxia, which might have influenced their performance on the 6MWT (Sivaranjini et al. 2010). The level of breathlessness experienced by participants in this study was very slight and none of them reported a smoking history prior to admission, which might explain the greater improvement in distance achieved on the 6MWT after hospitalisation.

The majority of participants in this study had HIV seropositivity. This might be a factor that influenced their performance on the 6MWT in relation to distance achieved when compared with the healthy SA cohort (Van Aswegen et al. 2010). It is known that exercise capacity in HIV positive subjects is lower compared with healthy populations when assessed with the 6MWT (Oursler et al. 2006). Factors reported to have an influence on 6MWT distance findings in HIV cohorts are inspiratory muscle weakness, older age, current smoking status, airflow limitations, peak oxygen uptake and active TB (Pontotoring et al. 2010). Roos et al. (2014) reported a distance walked of 540.7 (11.05) m on the 6MWT for a relatively healthy HIV positive group with risk factors for cardiac disease attending an outpatient HIV clinic in Gauteng. In the present study, participants were discharged home because their conditions stabilised. It is known that a change of 30–50 m in distance walked on the 6MWT has clinical significance in the chronic cardiac and chronic pulmonary disease populations and positively impacts their survival. There is currently no data available for minimal clinically important differences in distance achieved with the 6MWT for patients with pulmonary TB or HIV seropositivity. The difference in distance achieved (42 m) on the 6MWT between hospitalisation and 6 months observed in this study might be clinically significant, but needs confirmation in a larger trial.

Participants found the additional one-leg stance and one-leg squat balance tests challenging to perform especially when vision was removed. Sanchez-Sellero and Soto-Valera (2016) found an association between three anti-TB therapy drugs and the development of vestibular and visual dysfunction. The drugs specified included ethambutol, pyrazinamide and isoniazid (Sanchez-Sellero & Soto-Valera 2016). Because none of our participants reported a diagnosed vestibular disorder at the time of recruitment, and were managed with these specific anti-TB drugs, it is possible that this had an influence on how they performed during the more challenging balance tests over the 6-month period. This phenomenon needs to be verified in a larger trial. Participants presented with good functional balance as none of them used an assistive device to walk during the TUG test or when performing the 6MWT. Only a small change in duration to test completion was detected during the 6-month period for the TUG test. Bohannon (2006) proposed reference values for the TUG test duration for healthy elderly people. The lowest age range reported on in that review was 60–69 years with a duration of 8.1 (range 7.1–9) s to test completion. In comparison, our younger TB cohort took longer to complete the TUG test at all three assessment points. Their performance is, however, comparable to that of a large cohort of ICU survivors who completed the TUG in 9.1 s at 3 months post-discharge (Denehy et al. 2014). It is possible that participants in the current study suffered from more sarcopenia than the healthy elderly group, and thus the longer duration observed for completion of the TUG test.

## Conclusion

Findings from this feasibility pilot study suggest significant budget implications to ensure the success of a prospective observational and longitudinal trial as additional testing equipment would need to be purchased, more research staff would need to be employed and trained to recruit participants from multiple test sites and to conduct follow-up assessments at community level. Additional travel costs would also need to be considered. Recovery of physical function in this small cohort seemed to be affected by neuromusculoskeletal parameters (muscle architecture, mass and power; exercise capacity, and complex balancing activities) more than by respiratory symptoms particularly between 6 weeks and 6 months of anti-TB therapy.

### Recommendations

The abnormalities detected in rectus femoris and vastus lateralis muscle composition and limitations observed in general muscle power, complex balance activities and exercise capacity in this small cohort of patients on anti-TB therapy motivate for further exploration in longitudinal trials, provided that adequate funding can be secured. Findings from such longitudinal trials can be used to lobby for extension of rehabilitation services provided to this patient population, beyond hospital discharge, to optimise their physical and functional recovery at completion of anti-TB therapy.
